# Prevalence of strongyloidiasis in Peru: systematic review and meta-analysis

**DOI:** 10.1186/s12879-021-06441-9

**Published:** 2021-08-04

**Authors:** Sonia Ortiz-Martínez, José-Manuel Ramos-Rincón, María-Esteyner Vásquez-Chasnamote, Olga-Nohelia Gamboa-Paredes, Katty-Madeleine Arista-Flores, Luis-Alfredo Espinoza-Venegas, Eva de-Miguel-Balsa, Viviana-Vanessa Pinedo-Cancino, Miguel Górgolas-Hernández-Mora, Martín Casapía-Morales

**Affiliations:** 1 Medical Practice El Ballestero, Health Service of Castilla La Mancha, Albacete, Spain; 2 Clinical Medicine Department, University Miguel Hernández of Elche, Ctra N-332, 03550 Sant Joan d’Alacant, Alicante, Spain; 3 Internal Medicine Service, General University Hospital of Alicante-ISABIAL, Alicante, Spain; 4 Natural Resources Research Center. National University of the Peruvian Amazon, Iquitos, Peru; 5 Research Assistant. Amazon Rainforest Civil Association, Iquitos, Peru; 6 Infectious Diseases and Tropical Medicine Service, Loreto Regional Hospital, Iquitos, Peru; 7 Intensive Care Unit, General University Hospital of Elche, Alicante, Spain; 8 Molecular Biology and Immunology Laboratory of the Specialized Unit of LIPNAA-CIRNA, National University of the Peruvian Amazon, Iquitos, Peru; 9 Infectious Disease Division, University Hospital Foundation Jiménez Díaz, Madrid, Spain; 10 Medicine Department, Autonomous University of Madrid, Madrid, Spain; 11 Medical Department, Amazon Rainforest Civil Association, Iquitos, Peru; 12 School of Medicine, National University of the Peruvian Amazon, Iquitos, Peru

**Keywords:** *Strongyloides stercoralis*, Serology, Seroprevalence, Prevalence, Peru

## Abstract

**Background:**

Strongyloidiasis is a disease of great public health significance, caused by the parasitic nematodes *Strongyloides stercoralis*, *Strongyloides fuelleborni*, and *Strongyloides fuelleborni* subsp*. kellyi*. This systematic review and meta-analysis aimed to assess the prevalence of *Strongyloides stercoralis* infection in Peru.

**Methods:**

The review was based on a literature search in PubMed, SciELO and Google Scholar using the key words or root words “strongyl*” AND “Peru” on 15 July 2020. Eligible studies were published from 1 January 1981 to 15 July 2020 and written in English, Spanish, Italian, or French.

**Results:**

We included 21 papers in the analysis. Studies were heterogeneous in terms of study population and diagnostic methods (e.g. Baermann technique, agar, Dancescu or charcoal cultures, serology, string capsule). Prevalence of *S. stercoralis* ranged from 0.3 to 45%. The pooled proportion of *Strongyloides* in the general population was 7.34% (95% CI 4.97 to 10.13%). Half the studies were designed to detect parasites in general. In studies designed to detect *S. stercoralis*, the most widely used diagnostic method was the Baermann technique.

**Conclusion:**

Prevalence of *S. stercoralis* in Peru was high but varied by geographic area, techniques for stool examination, and participant characteristics.

## Background

Strongyloidiasis is a chronic, neglected disease, caused by the nematodes *Strongyloides stercoralis, Strongyloides fuelleborni*, and *Strongyloides fuelleborni* subsp. *kellyi* [1]. This soil-transmitted helminthiasis is believed to affect around 370 million people worldwide [2, 3], but prevalence could actually be much higher; a 2017 study estimated global prevalence at 8.1%, or 613.9 million people [4]. *Strongyloides stercoralis* is endemic to tropical and subtropical regions, but it can be found anywhere with an increased risk of fecal contamination due to poor sanitation, inadequate water supply, or other factors [1, 2]. Infection rates and risk vary among different population groups [5]. *S. stercoralis* is the most widely spread species and the only one capable of autoinfection. Untreated, the infection can persist for years to decades and cause considerable morbidity and mortality [6]. Ivermectin, a broad spectrum antiparasitic drug that is used frequently in humans, is the most effective drug against *S. stercoralis.*

More broadly, intestinal parasitosis is a public health problem in Peru, with an estimated one out of every three Peruvians carrying at least one parasite in their intestines. Studies have shown that strongyloidiasis is highly prevalent in Peruvian rainforest communities but less so in the mountains and coastal areas [7, 8]. Currently there are no national policies that promote the eradication of *S. stercoralis* in children or adults in Peru [9].

The Ministry of Health in Peru reviewed the cross-sectional prevalence studies of *S. stercoralis* infection from different areas of the country between 1981 and 2001 [10]. Mean prevalence was 6.6%, with variations by location and diagnostic methods. Despite the high prevalence of the infection, there is limited knowledge of its epidemiology and sero-epidemiology [11].

The difficulty in diagnosing *S. stercoralis* lies in the absence of a reliable gold standard diagnostic test. Although in some studies the Baermann technique has been shown to be superior to agar plate culture (APC), in others APC is more sensitive than Dancescu or charcoal cultures, the Baermann technique and spontaneous sedimentation in tube technique (SSTT). In contrast, the method with the lowest sensitivity is direct examination. Serology overestimates the burden of disease in endemic countries due to the cross-reactivity with other nematode infections [7, 12].

The sensitivity of diagnostic methods improves with a larger number of stool samples collected; three stool samples can increase the detection sensitivity by up to 7%, while seven stool samples yield a sensitivity of almost 100% [1, 13].

This systematic literature review and meta-analysis aimed to assess the prevalence of *S. stercoralis* infection in Peru.

## Methods

We performed an electronic search in PubMed and SciELO on 15 July 2020, using the following key words or root words, grouped into two main concepts: “strongyl*” AND “Peru”. Results were restricted to studies published from 1 January 1981 to 15 July 2020; performed in humans; and written in English, Spanish, Italian, or French. We sought to identify additional records through backward reference searching and electronic searches for grey literature (Google and Google Scholar).

We assessed surveys, notes, analyses, and epidemiological reports on the prevalence of intestinal protozoa and helminths in general or strongyloidiasis in particular. Two authors screened the titles and abstracts for relevance, retrieving the full texts of all eligible or potentially eligible articles. Data on the prevalence of *S. stercoralis* were collected regardless of the population characteristics (children, adults, immunocompromised patients, etc.). Study characteristics were also collected, including population, study design, diagnostic procedure, and type of fecal examination technique (e.g. modified Baermann technique).

We performed a proportion meta-analysis of prevalence estimates, using the Stuart-Ord (inverse double arcsine square root) method (random-effects model) to calculate the 95% coefficient intervals and create the forest plots. Heterogeneity was analyzed using the I^2^ statistic [14]. Publication bias was investigated with funnel plot and confirmed with Egger’s test. Meta-analyses were performed with StatsDirect Statstical Software v. 3.3.4 (StatsDirect Ltd., Merseyside, UK).

The prevalence meta-analysis was presented according to three population groups: general population, children, and adults.

## Results

**Fig. 1:**
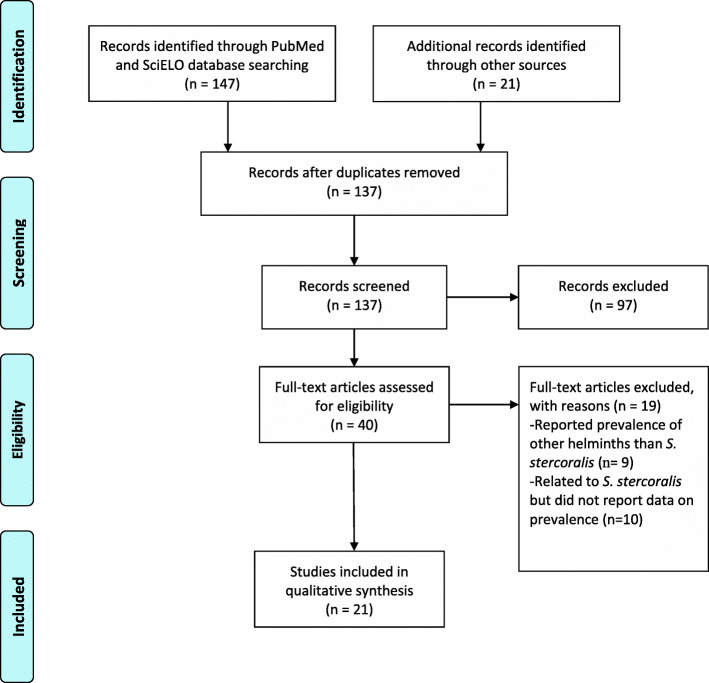
Flow chart for study selection

The electronic search in PubMed and SciELO yielded 147 records, and 21 papers were identified through additional searches. After screening the titles and abstracts, we examined the full text of 42 potentially relevant papers, excluding 21 that reported the prevalence of other helminthic infections or did not report prevalence data. The remaining 21 studies were included. Figure 1 shows the flow chart for study selection.

All studies used a cross-sectional, descriptive, observational design, except one systematic review, performed by the Peruvian Ministry of Health, which collected 294 parasite prevalence studies between 1981 and 2001 [10].

The study populations were very heterogeneous: three papers included the adult population (over 15 years old) [15–17], eight were in children [8, 9, 18–23], and nine studied both adults and children [7, 10, 11, 24–29]. One study included only people infected with HIV [15] and another only pregnant women [17].

Most studies described symptomatology. In one study, participants with strongyloidiasis were asymptomatic [20], while in another, authors mentioned only anemia [27]. Six studies did not discuss symptoms [7, 11, 22, 25, 28, 30].

Half the studies were designed to detect parasites in general. Among those designed specifically to study *S. stercoralis*, 10 used the Baermann technique for diagnosis [7, 9–11, 15–17, 21, 25, 29]. Seven studies used agar, Dancescu or charcoal cultures [7, 9–11, 17, 21, 29]; two performed specific serological tests [11, 17]; and one employed a string capsule/enterotest [30]. Other diagnostic techniques used to detect the presence of larvae in stool samples included direct smear of feces in saline–Lugol iodine stain, spontaneous tube sedimentation, formalin-ethyl acetate concentration, and Harada–Mori filter paper culture.

The studies in which culture and Baermann’s technique were used as diagnostic methods reported higher prevalence than those that only used the direct examination or Kato-Katz [22, 26].

Regarding sample collection, most studies collected a single stool sample; four studies used more than one [15, 19, 23, 24]. Investigators in four studies also collected blood samples, in two cases for performing *S. stercoralis* serology [11, 17], in two for assessing anemia [17, 27], and in one for evaluating eosinophilia [22].

Table 1 presents a summary of the characteristics of included studies [7–27]. Estimated prevalence of strongyloidiasis ranged from 0.3 to 45% [7, 26], depending on the geographic study area, the techniques used for stool examination, and participant characteristics (e.g. those with diarrhea versus asymptomatic individuals); overall rates were stable over the study period.

**Table 1:** Peruvian studies of *Strongyloides stercoralis* infection

Study ID	Setting (department) [altitude]	Population	Diagnostic procedure	Prevalence estimate
Huaroto Sedda 1990 [30]	National Hospital “Edgardo Rebagliati Martins” (Lima) [160 m]	1511 patients in Gastroenterology Service	– String capsule – Enterotest (1 sample)	2.4%
Rodriguez 1991 [18]	Tarapoto, Amazon basin (San Martin) [141 m]	110 preschool children	– Direct smear – Faust floaty concentration – Willis floating – Graham’s tape	16%
Egido 2001 [24]	Clinical Hospital, Puerto Maldonado, Amazon Basin (Madre de Dios) [139 m]	1133 outpatients with diarrhea (children and adults)	– Direct fecal smears with saline solution and Lugol stain (3 samples)	19.5%
Marcos Raymundo 2002 [19]	Rural survey, province of Jauja (Junin) [3391 m]	188 children (1–16 years old)	– Spontaneous tube sedimentation technique – Formalin-ether concentration – Rapid sedimentation technique, modified by Lumbreras	1.5%
Marcos 2002 [16]	Hospital cross-sectional study, Iquitos (Loreto) [100 m]	41 adults (20 from Military Hospital, 21 from Regional Hospital)	– Direct microscopy – Kato-Katz technique – Spontaneous tube sedimentation technique – Modified Baermann method	45 and 4.8%
Marcos 2003 [25]	Community survey, rural and urban populations of Sandia (Puno) [2135 m]	72 children and adults	– Direct microscopy – Kato-Katz technique – Spontaneous tube sedimentation technique – Modified Baermann method	1.4%
Ministry of Health 2003 [10]	Cross-sectional studies (countrywide)	294 studies and 214,199 people	– Various	6.6%
Ibañez 2004 [20]	Survey in rural community, Chancay district, Huaral province (Lima) [43 m]	1049 children (6–15 years old)	– Direct examination – Spontaneous tube sedimentation – Rapid sedimentation technique modified by Lumbreras – Kato-Katz technique – Baermann method modified by Lumbreras	0.8%
Lau Chong 2005 [7]	Survey in rural community, Peruvian Amazon, Oxapampa province (Pasco) [NA] [1814 m]	129 children and adults	– Simple direct smear – Spontaneous tube sedimentation – Baermann method modified by Lumbreras – Dancescu culture – Agar plate culture technique	38.5%
Yori PP 2006 [11]	Survey in rural community on Nanay River, Amazon Basin (Loreto) [100 m]	908 children and adults	– Direct smear – Baermann method – Simple sedimentation – ELISA positive	8.7% 72% seroprevalence
Garcia 2006 [15]	Cayetano Heredia National Hospital, Lima (Lima) [160 m]	217 patients with HIV/AIDS	– Direct examination – Kato-Katz technique – Spontaneous tube sedimentation – Baermann method modified by Lumbreras – Rapid sedimentation technique modified by Lumbreras – Ziehl Neelsen stain	6%
Crotti 2007 [26]	Chacas Hospital (Lima) [3300–3500 m]	91 patients (38 children + 53 adults)	– Microscopic observations (direct and after formalin-ether concentration) – Giemsa permanent stain	0.3%
Natividad-Carpio 2007 [8]	Community survey, Chancay district, Huaral province (Lima) [161 m]	173 children (2–20 years-old)	– Direct examination – Spontaneous tube sedimentation – Rapid sedimentation technique modified by Lumbreras – Kato-Katz technique – Baermann method, modified by Lumbreras	1.1%
Machicado 2012 [21]	Rural survey, Tambopata province (Madre de Dios). Peruvian Rainforest [200 m]	73 children (2–20 years old)	– Spontaneous tube sedimentation – Kato-Katz technique – Modified Baermann method – Agar plate culture – Harada-Mori culture – Direct smear examination	16%
Cabada 2014 [27]	Rural survey following deworming campaign, southern Peruvian Amazon (Madre de Dios) [600 m]	290 members of the Matsiguenga ethnic group	– Direct examination – Rapid sedimentation – Kato-Katz technique	5.6%
Cabada 2014 [22]	Rural communities survey around Cusco [3300–3500 m]	227 children (3–12 years old)	– Direct examination – Rapid sedimentation – Kato-Katz technique	0.9%
Cabada 2016 [23]	Rural survey communities around Cusco (Cusco) [3300–3500 m]	1230 children (3–16 years old)	– Lumbreras rapid sedimentation tests – Kato-Katz technique (3 samples)	2%
Garaycochea 2018 [28]	Provinces of Huaral, Oyón, Yauyos and Huarochirí. (Lima) [188–3600–2800-3100 m]	359 (children < 5 years old)	– Direct sedimentation methods – Heidenhain’s iron hematoxylin smear test – Modified Ziehl Neelsen – Graham’s method	6.8%
Morales 2019 [29]	Community survey, rural population around Cusco: Quellouno [800 m] and Limatambo [2554 m] [3300 m]	462 participants (children and adults)	– Baermann’s method – Agar plate culture – Sedimentation tests (1 sample)	24.5%* 26.4%** low altitude 18.6 high altitude
Errea 2019 [9]	Rural community surveys in Padre Cocha (Amazon Basin) (Loreto) [100 m]	124 children	– Direct smear analysis – Kato-Katz technique – Spontaneous tube sedimentation – Baermann’s method – Agar plate culture (1 sample)	10.5%
Ortiz-Martínez 2020 [17]	Survey, urban and periurban Iquitos, Peruvian Amazon (Loreto) [100 m]	300 pregnant women (adults)	– Baermann’s method – Charcoal culture – Kato-Katz technique – ELISA (1 sample)	10% 30% seroprevalence

The highest prevalence (45%) was reported in 20 patients attended in the Military Hospital in Iquitos [16]. The study with the second highest prevalence (38.5%) took place in the Native Community of Nagazú in the Pasco region of the Peruvian Amazon, a central rainforest region recognized as endemic to *S. stercoralis* [7]. Other studies showed high prevalence in the Cusco region (24.5%) and in participants with diarrhea in Puerto Maldonado (19.5%) [24, 29]. Morales et al. reported a higher prevalence of *Strongyloides* infection at low altitudes (26.4%) compared to mountainous regions (18.6%) [29]. Low prevalence, of 0.3 to 1.5% of the sample population, was also observed in Chacas and in children living in Jauja (Junin department) [8, 19, 20, 22, 25, 26].

Most studies discussed coinfection with various helminths. In a study in Puerto Maldonado (Peruvian Amazon), nearly half the participants (47.1%) infected with *S. stercoralis* were coinfected with *Ancylostoma duodenale* [24]. In Tarapoto [18], 42% of total participants had parasitic coinfections; the most common pair was *Ascaris lumbricoides* and *Trichuris trichura*. In four provinces of the department of Lima [28], biparasitosis was 32.7%; authors did not report the most common association. The study reporting the fewest mixed parasitic infections (8.3%) involved 217 HIV patients at the Cayetano Heredia National Hospital in Lima [15].

**Fig. 2:**
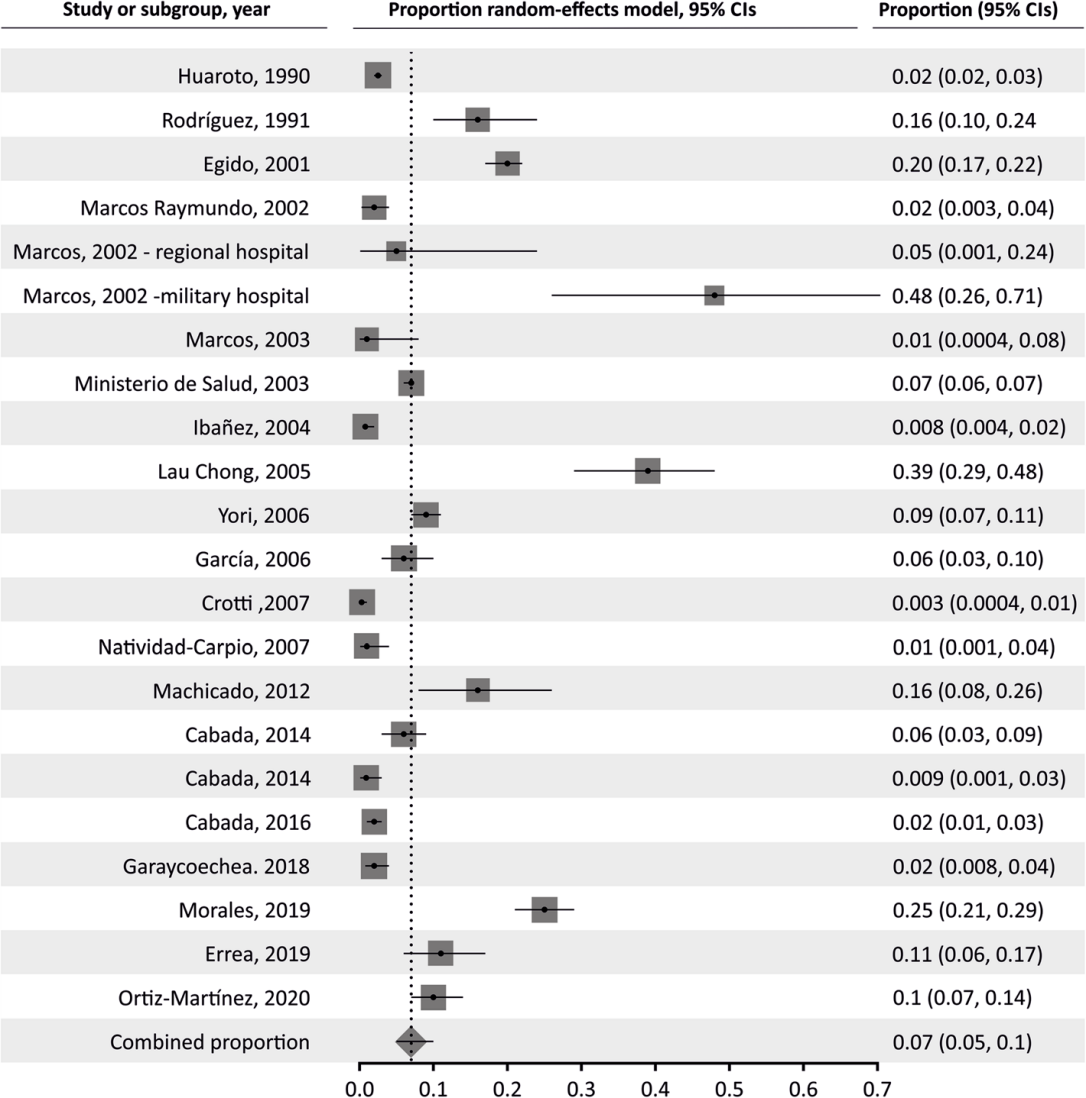
Forest plot of pooled prevalence of *Strongyloides* infection in general population

**Fig. 3:**
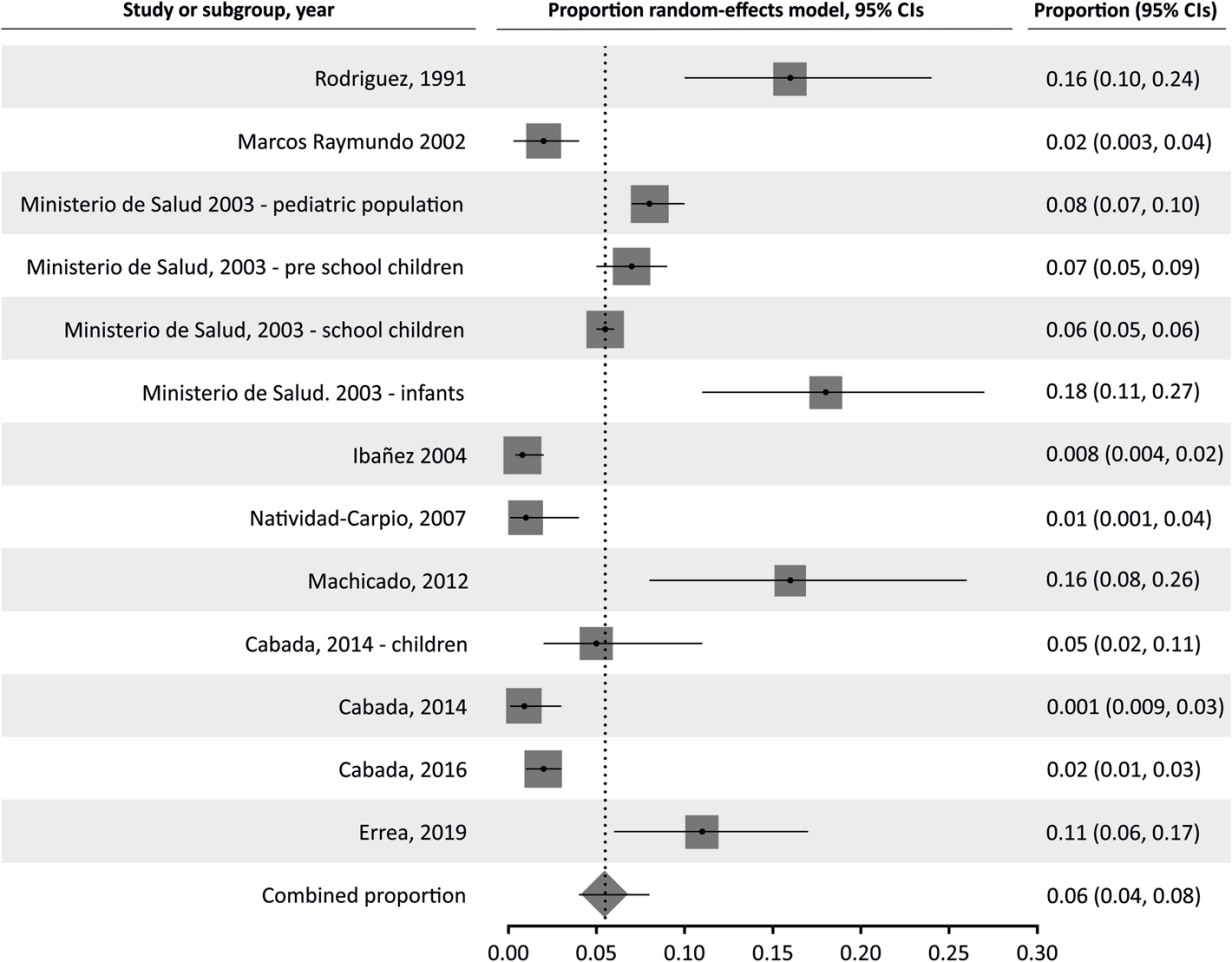
Forest plot of pooled prevalence of *Strongyloides* infection in children

**Fig. 4:**
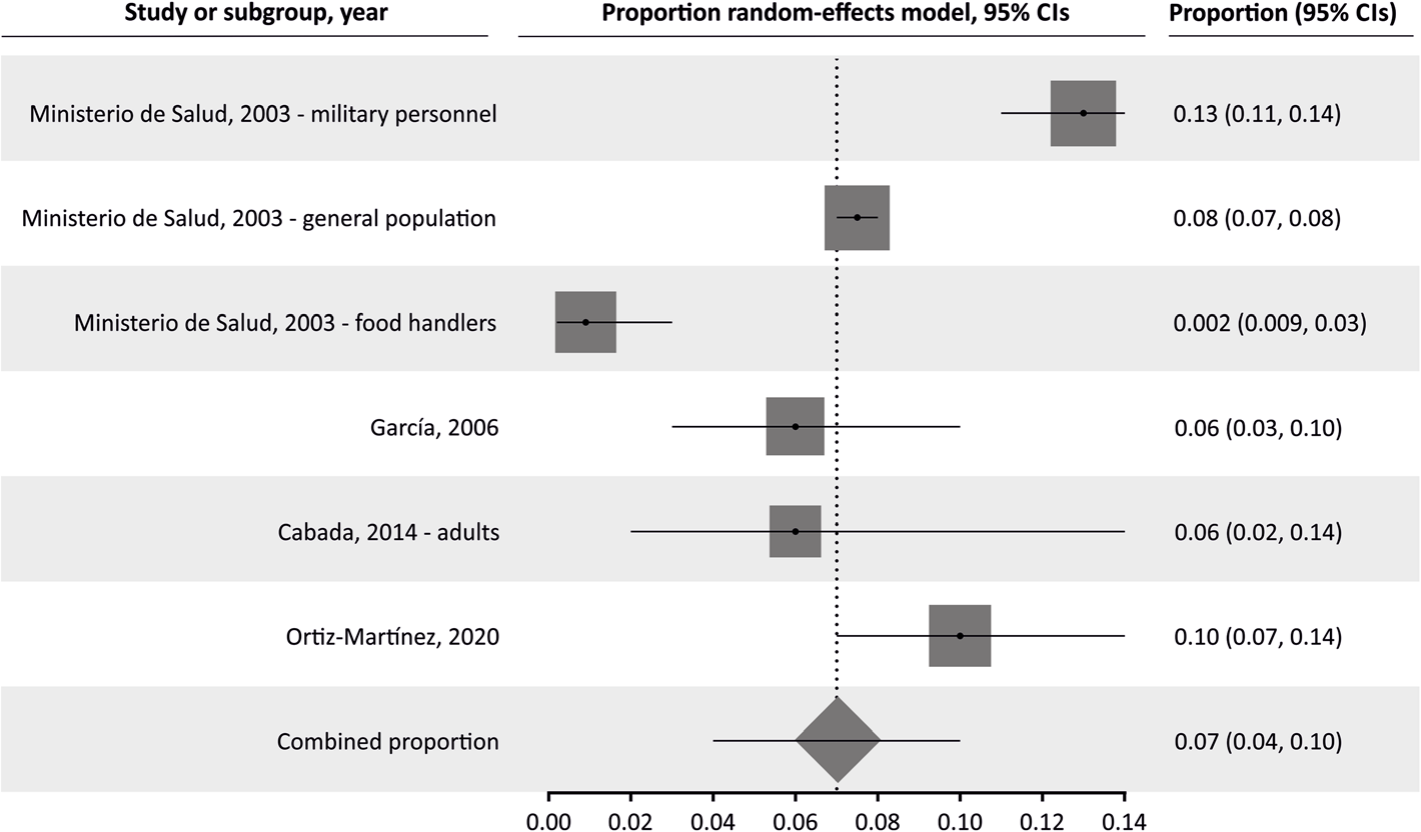
Forest plot of pooled prevalence of *Strongyloides* infection in adults

The pooled proportion of *Strongyloides* in the general population was 7.34% (95% CI 4.97 to 10.13%; I^2^ 97.51%). Table 2 summarizes the results of pooled prevalence in pediatric and adult populations, assuming a random-effects model. As expected, we found high heterogeneity in general and adult populations, although not in studies in children. Figures 2, 3 and 4 summarize the prevalence in general, pediatric, and adult populations.

**Table 2:** Pooled prevalence analysis of *Strongyloides* infection in different population groups

	Pooled prevalence	95% confidence intervals	N studies	N participants	I^**2**^, %	Egger’s test
General pop.	7.34	4.97–10.13	16	51,094	97.51	0.315
Children	5.59	3.67–7.88	13	15,793	94.70	1.301
Adults	6.99	4.24–103.5	6	11,693	95.00	−0.379

## Discussion

The prevalence of *S. stercoralis* varies by geographical area. Prevalence in the rainforest is 18.5%; in the mountains, 2.2% (2300–3500 m altitude) to 4.3% (3500–4000 m altitude); and on the coast, 3.0% [10]. In another study *Strongyloides* infection was more common at low altitudes (26.4%) compared to high altitudes (18.6%) [29]. Among the studies that collected several samples, the highest prevalence observed was 19.5% [24]. In contrast, two studies reported very low prevalence: 1.5 and 2% [19, 23], despite having increased the diagnostic yield with multiple samples. These differences are probably due to geographical factors, as the first study took place in the Peruvian Amazon and the other two at more than 3300 m altitude. The filariform larvae of *S. stercoralis* are known to survive for about 2 weeks at temperatures between 8 °C and 40 °C, but they cannot withstand excessive aridity or humidity [31]. This may be one reason why *Strongyloides* is more prevalent in the rainforest than in the mountains. On the other hand, mountain inhabitants may travel to the rainforest on a seasonal basis, which could explain why the prevalence is higher at 3500 m compared to 2300 m.

Estimating the prevalence of *S. stercoralis* in Peru is complicated by the diversity of diagnostic methods used. Not all methods have the same ability to detect it. It would be necessary to systematize the studies using several diagnostic procedures (Baermann method, charcoal culture, and probably serology) to have a more accurate estimate of the true prevalence.

A study designed for diagnosing *S. stercoralis* infection in a rural community in Iquitos [11] showed that 8.7% of the stool samples were positive using one of three diagnostic methods, although the sensitivity differed between them: direct examination (sensitivity 37.7%), Baermann method (40.5%), or simple sedimentation (79.7%). APC showed a sensitivity of just 60.9% due to an exuberant growth of fungi in 35% of the samples, which prevented interpretation. Enzyme-linked immunosorbent assay (ELISA) was positive in 72% of the blood samples, and the negative predictive value of the serology was 98%. In another study from the same region, the prevalence of *S. stercoralis* infection was 10% using the Baermann method, charcoal culture and ELISA for diagnosis. In this case, the sensitivity of the serology was 63.3% and the negative predictive value, 94.4% [17].

Machicado et al. [21] calculated the percentage of samples positive for *S. stercoralis* with each diagnostic method, observing that APC was the most sensitive method (81%), followed by the modified Baermann technique and SSTT (75%). The sensitivity of the Harada-Mori culture was much lower at 19%, while the direct smear or Kato-Katz had 0% sensitivity. In the same study, the authors found no difference in the number of diagnosed cases of *A. lumbricoides*, *T. trichura*, and hookworm, using either the Kato-Katz or spontaneous sedimentation in tube technique. Thus, the SSTT could be a good diagnostic method for *S. stercoralis* as well as other helminths.

Techniques such as Baermann’s or APC are cumbersome and time-consuming. Multiple samples must be collected on different days to improve the detection rate because of the irregular excretion pattern of *S. stercoralis* larvae, especially for low-intensity infections. Another drawback is the need for fresh and non-refrigerated stool samples.

Serology is useful, but this method could overestimate the prevalence of the disease due to cross-reactivity with other nematode infections; moreover, distinguishing recent infections from past (and cured) ones is not straightforward [12].

The prevalence of strongyloidiasis varies according to the characteristics of the study population. The high prevalence in Marcos et al.’s study (45%) can be attributed to the military population, which was exposed to untreated drinking water, lack of sanitation, overcrowding, and environmental risks during the course of their work [16]. These same conditions are found in rural communities where the studies with the highest prevalence take place [7, 21, 29]. The exception is the study by Egido et al. [24], where the population presents diarrhea; in the patients with symptoms (diarrhea) the prevalence of *S. stercoralis* as well as other pathogens was higher.

There is only one study carried out in pregnant women, showing a prevalence of 10%. Similar prevalence (10.5%) was observed in children in the same geographical area [9] and in the study by Yori et al. [11] in the general population (8.7%). This suggests that prevalence in pregnant women may be similar to the rest of the population in the same area, but more studies would be necessary to confirm. Given that pregnant women are especially vulnerable to disseminated strongyloidiasis and hyperinfection due to the immunosuppression of pregnancy itself, chronic nutritional deficiencies, and the occasional use of corticosteroids for fetal lung maturation, it would be important to implement active search and control programs for *S. stercoralis* during antenatal visits.

Our review is limited by the relatively small number of included studies, the lack of standard definitions, the use of different diagnostic techniques, and the study of multiparasitosis.

## Conclusions

Prevalence of *S. stercoralis* in Peru ranged from 0.3 to 45%, with variations by geographic study area, stool examination techniques, and participant characteristics. Small-scale prevalence estimates within a country do not accurately reflect the variation in distribution. They are not necessarily representative of a country as a whole. Therefore, it would be necessary to establish a specific diagnostic protocol *for S. stercoralis* together with adequate sampling and statistical analysis to estimate the real prevalence of strongyloidiasis in Peru. This would be the starting point for the development of an integrated soil-transmitted helminthiasis control program.

## Data Availability

The datasets generated are available from the corresponding author on reasonable request.

## Abbreviations

APC: agar plate culture
ELISA: Enzyme-Linked ImmunoSorbent Assay
SSTT: Spontaneous sedimentation in tube technique
